# Strategies for single-shot discrimination of process matrices

**DOI:** 10.1038/s41598-023-30191-0

**Published:** 2023-02-21

**Authors:** Paulina Lewandowska, Łukasz Pawela, Zbigniew Puchała

**Affiliations:** Institute of Theoretical and Applied Informatics, Polish Academy of Sciences, ul. Bałtycka 5, 44-100 Gliwice, Poland

**Keywords:** Quantum information, Information theory and computation

## Abstract

The topic of causality has recently gained traction quantum information research. This work examines the problem of single-shot discrimination between process matrices which are an universal method defining a causal structure. We provide an exact expression for the optimal probability of correct distinction. In addition, we present an alternative way to achieve this expression by using the convex cone structure theory. We also express the discrimination task as semidefinite programming. Due to that, we have created the SDP calculating the distance between process matrices and we quantify it in terms of the trace norm. As a valuable by-product, the program finds an optimal realization of the discrimination task. We also find two classes of process matrices which can be distinguished perfectly. Our main result, however, is a consideration of the discrimination task for process matrices corresponding to quantum combs. We study which strategy, adaptive or non-signalling, should be used during the discrimination task. We proved that no matter which strategy you choose, the probability of distinguishing two process matrices being a quantum comb is the same.

## Introduction

The topic of causality has remained a staple in quantum physics and quantum information theory for recent years. The idea of a causal influence in quantum physics is best illustrated by considering two characters, Alice and Bob, preparing experiments in two separate laboratories. Each of them receives a physical system and performs an operation on it. After that, they send their respective system out of the laboratory. In a causally ordered framework, there are three possibilities: Bob cannot signal to Alice, which means the choice of Bob’s action cannot influence the statistics Alice records (denoted by $A \prec B$), Alice cannot signal to Bob ($B \prec A$), or neither party can influence the other (*A*||*B*). A causally neutral formulation of quantum theory is described in terms of quantum combs^1^.

One may wonder if Alice’s and Bob’s action can influence each other. It might seem impossible, except in a world with closed time-like curves (CTCs)^2^. But the existence of CTCs implies some logical paradoxes, such as the grandfather paradox^3^. Possible solutions have been proposed in which quantum mechanics and CTCs can exist and such paradoxes are avoided, but modifying quantum theory into a nonlinear one^4^. A natural question arises: is it possible to keep the framework of linear quantum theory and still go beyond definite causal structures?

One such framework was proposed by Oreshkov, Costa and Brukner ^5^. They introduced a new resource called a process matrix—a generalization of the notion of quantum state. This new approach has provided a consistent representation of correlations in casually and non-causally related experiments. Most interestingly, they have described a situation that two actions are neither causally ordered and one cannot say which action influences the second one. Thanks to that, the term of causally non-separable (CNS) structures started to correspond to superpositions of situations in which, roughly speaking, Alice can signal to Bob, and Bob can signal to Alice, jointly. A general overview of causal connection theory is described in Ref.^6^.

The indefinite causal structures could make a new aspect of quantum information processing. This more general model of computation can outperform causal quantum computers in specific tasks, such as learning or discriminating between two quantum channels^7–9^. The problem of discriminating quantum operations is of the utmost importance in modern quantum information science. Imagine we have an unknown operation hidden in a black box. We only have information that it is one of two operations. The goal is to determine an optimal strategy for this process that achieves the highest possible probability of discrimination. For the case of a single-shot discrimination scenario, researchers have used different approaches, with the possibility of using entanglement in order to perform an optimal protocol. In Ref.^10^, Authors have shown that in the task of discrimination of unitary channels, the entanglement is not necessary, whereas for quantum measurements^11–13^, we need to use entanglement. Considering multiple-shot discrimination scenarios, researchers have utilized parallel or adaptive approaches. In the parallel case, we establish that the discrimination between operations does not require pre-processing and post-processing. One example of such an approach is distinguishing unitary channels^10^, or von Neumann measurements^14^. The case when the black box can be used multiple times in an adaptive way was investigated by the authors of Refs.^15,16^, who have proven that the use of adaptive strategy and a general notion of quantum combs can improve discrimination. It is worth noting that the problem of discrimination between process matrices is a direct analogy to the discrimination task of quantum states or channels. In general, the discrimination task of quantum objects helps study the geometry of sets of these objects by inducing the trace norm (for quantum states) or the diamond norm (for quantum channels and measurements). These norms constitute the basis for quantum capacity theory^17^, quantum error correction^18^ or quantum cryptography^19^.

In this work, we study the problem of discriminating process matrices in a single-shot scenario. We obtain that the probability of correct distinction process matrices is strictly related to the Holevo–Helstrom theorem for quantum channels. Additionally, we write this result as a semidefinite program (SDP) which is numerically efficient. The SDP program allows us to find an optimal discrimination strategy. We compare the effectiveness of the obtained strategy with the previously mentioned strategies. The problem gets more complex in the case when we consider the non-causally ordered framework. In this case, we consider the discrimination task between two process matrices having different causal orders.

This paper is organized as follows. In “Mathematical preliminaries” section we introduce necessary mathematical framework. “Process matrices” section is dedicated to the concept of process matrices. “Discrimination task” section presents the discrimination task between pairs of process matrices and calculate the exact probability of distinguishing them. Some examples of discrimination between different classes of process matrices are presented in “Discrimination between different classes of process matrices” section. In “Free process matrices” section, we consider the discrimination task between free process matrices, whereas in “Process matrices representing quantum combs” section we consider the discrimination task between process matrices being quantum combs. In “[Sec Sec8]” section, we show a particular class of process matrices having opposite causal structures which can be distinguished perfectly. Finally, “SDP program for calculating the optimal probability of process matrices discrimination” and “Distance between process matrices” sections are devoted to semidefinite programming, thanks to which, among other things, we obtain an optimal discrimination strategy. In “Convex cone structure theory” section, we analyze an alternative way to achieve this expression using the convex cone structure theory. Concluding remarks are presented in the final “Conclusion and discussion” section. In the Supplementary Materials, we provide technical details about the convex cone structure.

## Mathematical preliminaries

Let us introduce the following notation. Consider two complex Euclidean spaces and denote them by $\mathcal {X}, \mathcal {Y}$. By $\text{L}(\mathcal {X}, \mathcal {Y})$ we denote the collection of all linear mappings of the form $A: \mathcal {X}\rightarrow \mathcal {Y}$. As a shorthand put $\text{L}(\mathcal {X}) :=\text{L}(\mathcal {X}, \mathcal {X}).$ By $\text{Herm}(\mathcal {X})$ we denote the set of Hermitian operators while the subset of $\text{Herm}(\mathcal {X})$ consisting of positive semidefinite operators will be denoted by $\text{Pos}(\mathcal {X})$. The set of quantum states, that is positive semidefinite operators $\rho$ such that ${{\,\text{tr}\,}}\rho = 1$, will be denoted by $\Omega (\mathcal {X})$. An operator $U \in \text{L}\left( \mathcal {X}\right)$ is unitary if it satisfies the equation $U U^\dagger = U^\dagger U = \mathbbm {1}_\mathcal {X}$. The notation $\text{U}\left( \mathcal {X}\right)$ will be used to denote the set of all unitary operators. We will also need a linear mapping of the form $\Phi : \text{L}(\mathcal {X}) \rightarrow \text{L}(\mathcal {Y})$ transforming $\text {L}(\mathcal {X})$ into $\text {L}(\mathcal {Y})$. The set of all linear mappings is denoted $\text{M}(\mathcal {X}, \mathcal {Y})$. There exists a bijection between set $\text{M}(\mathcal {X}, \mathcal {Y})$ and the set of operators $\text{L}(\mathcal {Y}\otimes \mathcal {X})$ known as the Choi^20^ and Jamiołkowski^21^ isomorphism. For a given linear mapping $\Phi _M: \text{L}(\mathcal {X}) \rightarrow \text{L}(\mathcal {Y})$ corresponding Choi matrix $M \in \text{L}(\mathcal {Y}\otimes \mathcal {X})$ can be explicitly written as

1 $$\begin{aligned} M :=\sum _{i,j = 0}^{\dim (\mathcal {X}) - 1} \Phi _M(|i\rangle \! \langle j|) \otimes |i\rangle \! \langle j|. \end{aligned}$$

We will denote linear mappings by $\Phi _M, \Phi _N, \Phi _R$ etc., whereas the corresponding Choi matrices as plain symbols: *M*, *N*, *R* etc. Let us consider a composition of mappings $\Phi _R = \Phi _N \circ \Phi _M$ where $\Phi _N: \text{L}(\mathcal {Z}) \rightarrow \text{L}(\mathcal {Y})$ and $\Phi _M: \text{L}(\mathcal {X}) \rightarrow \text{L}(\mathcal {Z})$ with Choi matrices $N \in \text{L}(\mathcal {Z}\otimes \mathcal {Y})$ and $M \in \text{L}(\mathcal {X}\otimes \mathcal {Z})$, respectively. Then, the Choi matrix of $\Phi _R$ is given by^22^

2 $$\begin{aligned} R = {{\,\text{tr}\,}}_{\mathcal {Z}} \left[ \left( \mathbbm {1}_\mathcal {Y}\otimes M^{T_\mathcal {Z}}\right) \left( N \otimes \mathbbm {1}_\mathcal {X}\right) \right] , \end{aligned}$$

where $M^{T_\mathcal {Z}}$ denotes the partial transposition of *M* on the subspace $\mathcal {Z}$. The above result can be expressed by introducing the notation of the link product of the operators *N* and *M* as

3 $$\begin{aligned} N * M :={{\,\text{tr}\,}}_{\mathcal {Z}} \left[ \left( \mathbbm {1}_\mathcal {Y}\otimes M^{T_\mathcal {Z}}\right) \left( N \otimes \mathbbm {1}_\mathcal {X}\right) \right] . \end{aligned}$$

Finally, we introduce a special subset of all mappings $\Phi$, called quantum channels, which are completely positive and trace preserving (CPTP). In other words, the first condition reads

4 $$\begin{aligned} (\Phi \otimes \mathcal {I_\mathcal {Z}})(X) \in \text{Pos}(\mathcal {Y}\otimes \mathcal {Z}), \end{aligned}$$

for all $X \in \text{Pos}(\mathcal {X}\otimes \mathcal {Z})$ and $\mathcal {I_\mathcal {Z}}$ is an identity channel acts on $\text{L}(\mathcal {Z})$ for any $\mathcal {Z}$, while the second condition reads

5 $$\begin{aligned} {{\,\text{tr}\,}}(\Phi (X)) = {{\,\text{tr}\,}}(X) \end{aligned}$$

for all $$X \in \text{L}(\mathcal {X})$$.

In this work we will consider a special class of quantum channels called non-signaling channels (or causal channels)^23,24^. We say that $\Phi _N: \text{L}(\mathcal {X}_I \otimes \mathcal {Y}_I) \rightarrow \text{L}(\mathcal {X}_O \otimes \mathcal {Y}_O)$ is a non-signaling channel if its Choi operator satisfies the following conditions

6 $$\begin{aligned} \begin{aligned}{}&{{\,\text{tr}\,}}_{\mathcal {X}_O} (N) = \frac{\mathbbm {1}_{\mathcal {X}_I}}{\dim (\mathcal {X}_I)} \otimes {{\,\text{tr}\,}}_{\mathcal {X}_O\mathcal {X}_1} (N), \\ {}&{{\,\text{tr}\,}}_{\mathcal {Y}_O} (N) = \frac{\mathbbm {1}_{\mathcal {Y}_I}}{\dim (\mathcal {Y}_I)} \otimes {{\,\text{tr}\,}}_{\mathcal {Y}_O\mathcal {Y}_1} (N). \end{aligned} \end{aligned}$$

It can be shown^25^ that each non-signaling channel is an affine combination of product channels. More precisely, any non-signaling channel $\Phi _N : \text{L}(\mathcal {X}_I \otimes \mathcal {Y}_I) \rightarrow \text{L}(\mathcal {X}_O \otimes \mathcal {Y}_O)$ can be written as

7 $$\begin{aligned} \Phi _N = \sum _i \lambda _i \Phi _{S_i} \otimes \Phi _{T_i} , \end{aligned}$$

where $\Phi _{S_i} : \text{L}(\mathcal {X}_I ) \rightarrow \text{L}(\mathcal {X}_O )$ and $\Phi _{T_i}: \text{L}(\mathcal {Y}_I) \rightarrow \text{L}( \mathcal {Y}_O)$ are quantum channels, $\lambda _i \in \mathbb {R}$ such that $\sum _i \lambda _i = 1$. For the rest of this paper, by $\textbf{NS}(\mathcal {X}_I \otimes \mathcal {X}_O \otimes \mathcal {Y}_I \otimes \mathcal {Y}_O)$ we will denote the set of Choi matrices of non-signaling channels.

The most general quantum operations are represented by quantum instruments^26,27^, that is, collections of completely positive (CP) maps $\left\{ \Phi _{M_i} \right\} _i$ associated to all measurement outcomes, characterized by the property that $\sum _i \Phi _{M_i}$ is a quantum channel.

We will also consider the concept of quantum network and tester^1,28^. We say that $\Phi _{R^{(N)}}$ is a deterministic quantum network (or quantum comb) if it is a concatenation of *N* quantum channels and $R^{(N)} \in \text{L} \left( \bigotimes _{i=0}^{2N-1} \mathcal {X}_i \right)$ fulfills the following conditions

8 $$\begin{aligned} \begin{aligned} R^{(N)}&\ge 0, \\ {{\,\text{tr}\,}}_{\mathcal {X}_{2k-1}} \left( R^{(k)} \right)&= \mathbbm {1}_{\mathcal {X}_{2k-2}} \otimes R^{(k-1)}, \end{aligned} \end{aligned}$$

where $R^{(k-1)} \in \text{L} \left( \bigotimes _{i=0}^{2k-3} \mathcal {X}_i \right)$ is the Choi matrix of the reduced quantum comb with concatenation of $k-1$ quantum channels, $k = 2,\ldots , N$. We remind that a probabilistic quantum network $\Phi _{S^{(N)}}$ is equivalent to a concatenation of *N* completely positive trace non increasing linear maps. Then, the Choi operator $S^{(N)}$ of $\Phi _{S^{(N)}}$ satisfies $0 \le S^{(N)} \le R^{(N)}$, where $R^{(N)}$ is Choi matrix of a quantum comb. Finally, we recall the definition of a quantum tester. A quantum tester is a collection of probabilistic quantum networks $\left\{ R_{i}^{(N)} \right\} _i$ whose sum is a quantum comb, that is $\sum _i R^{(N)}_i = R^{(N)}$, and additionally $\dim (\mathcal {X}_0) = \dim (\mathcal {X}_{2N-1}) = 1$.

We will also use the Moore–Penrose pseudo–inverse by abusing notation $X^{-1} \in \text{L}(\mathcal {Y}, \mathcal {X})$ for an operator $X \in \text{L}(\mathcal {X}, \mathcal {Y})$. Moreover, we introduce the vectorization operation of *X* defined by $|X \rangle \rangle = \sum _{i=0}^{\dim (\mathcal {X}) -1} (X |i\rangle ) \otimes |i\rangle$.

## Process matrices

This section introduces the formal definition of the process matrix with its characterization and intuition. Next, we present some classes of process matrices considered in this paper.

Let us define the operator $^{}_{\mathcal {X}}{Y}$ as

9 $$\begin{aligned} ^{}_{\mathcal {X}}{Y} = \frac{\mathbbm {1}_\mathcal {X}}{\dim (\mathcal {X})} \otimes {{\,\text{tr}\,}}_\mathcal {X}Y, \end{aligned}$$

for every $Y \in \text{L}(\mathcal {X}\otimes \mathcal {Z})$, where $\mathcal {Z}$ is an arbitrary complex Euclidean space. We will also need the following projection operator

10 $$\begin{aligned} L_V(W) = ^{}_{\mathcal {A}_O}{W} + ^{}_{\mathcal {B}_O}{W} - ^{}_{\mathcal {A}_O\mathcal {B}_O}{W} - ^{}_{\mathcal {B}_I\mathcal {B}_O}{W} + ^{}_{\mathcal {A}_O\mathcal {B}_I\mathcal {B}_O}{W} - ^{}_{\mathcal {A}_I\mathcal {A}_O}{W} + ^{}_{\mathcal {A}_O\mathcal {A}_I\mathcal {B}_O}{W}, \end{aligned}$$

where $$W \in \text{Herm}(\mathcal {A}_I\otimes \mathcal {A}_O \otimes \mathcal {B}_I \otimes \mathcal {B}_O)$$.

### Definition 1

We say that $W \in \text{Herm}(\mathcal {A}_I\otimes \mathcal {A}_O \otimes \mathcal {B}_I \otimes \mathcal {B}_O)$ is a process matrix if it fulfills the following conditions

11 $$\begin{aligned} W \ge 0, \,\,\, W = L_V(W), \,\,\ {{\,\text{tr}\,}}(W) = \dim (\mathcal {A}_O) \cdot \dim (\mathcal {B}_O), \end{aligned}$$

where the projection operator $L_V$ is defined by Eq. (10).

The set of all process matrices will be denoted by $\mathbf {W^{PROC}}$. In the upcoming considerations, it will be more convenient to work with the equivalent characterization of process matrices which can be found in Ref.^29^.

### Definition 2

We say that $W \in \mathbf {W^{PROC}}$ is a process matrix if it fulfills the following conditions

12 $$\begin{aligned} W\ge & {} 0,\nonumber \\ ^{}_{\mathcal {A}_I\mathcal {A}_O}{W}= & {} ^{}_{\mathcal {A}_O\mathcal {A}_I\mathcal {B}_O}{W},\nonumber \\ ^{}_{\mathcal {B}_I\mathcal {B}_O}{W}= & {} ^{}_{\mathcal {A}_O\mathcal {B}_I\mathcal {B}_O}{W}, W = ^{}_{\mathcal {B}_O}{W} +^{}_{\mathcal {A}_O}{W} - ^{}_{\mathcal {A}_O\mathcal {B}_O}{W},\nonumber \\ {{\,\text{tr}\,}}(W)= & {} \dim (\mathcal {A}_O) \cdot \dim (\mathcal {B}_O). \end{aligned}$$

The concept of process matrix can be best illustrated by considering two characters, Alice and Bob, performing experiments in two separate laboratories. Each party acts in a local laboratory, which can be identified by an input space $\mathcal {A}_I$ and an output space $\mathcal {A}_O$ for Alice, and analogously $\mathcal {B}_I$ and $\mathcal {B}_O$ for Bob. In general, a label *i*, denoting Alice’s measurement outcome, is associated with the CP map $\Phi _{M^{A}_i}$ obtained from the instrument $\left\{ \Phi _{M^{A}_i} \right\} _i$. Analogously, the Bob’s measurement outcome *j* is associated with the map $\Phi _{M^{B}_j}$ from the instrument $\left\{ \Phi _{M^{B}_j} \right\} _j$. Finally, the joint probability for a pair of outcomes *i* and *j* can be expressed as

13 $$\begin{aligned} p_{ij} = {{\,\text{tr}\,}}\left[ W \left( M_i^A \otimes M_j^B \right) \right] , \end{aligned}$$

where $W \in \mathbf {W^{PROC}}$ is a process matrix that describes the causal structure outside of the laboratories. The valid process matrix is defined by the requirement that probabilities are well defined, that is, they must be non-negative and sum up to one. These requirements give us the conditions present in Definitions 1 and 2.

In the general case, the Alice’s and Bob’s strategies can be more complex than the product strategy $M_i^A \otimes M_j^B$ which defines the probability $p_{ij}$ given by Eq. (13). If their action is somehow correlated, we can write the associated instrument in the following form $\left\{ \Phi _{ N_{ij}^{AB}}\right\}$. It was observed in Ref.^29^ that this instrument describes a valid strategy, that is

14 $$\begin{aligned} {{\,\text{tr}\,}}\left( W \sum _{ij} N_{ij}^{AB} \right) = 1, \end{aligned}$$

for all process matrix $W \in \mathbf {W^{PROC}}$ if and only if

15 $$\begin{aligned} \sum _{ij} N_{ij}^{AB} \in \textbf{NS} (\mathcal {A}_I \otimes \mathcal {A}_O \otimes \mathcal {B}_I \otimes \mathcal {B}_O). \end{aligned}$$

In this paper, we will consider different classes of process matrices. Initially, we define the subset of process matrices known as free objects in the resource theory of causal connection^30^. Such process matrices will be defined as follows.

### Definition 3

We say that $W^{A || B} \in \mathbf {W^{PROC}}$ is a free process matrix if it satisfies the following condition

16 $$\begin{aligned} W^{A||B} = \rho _{\mathcal {A}_I \mathcal {B}_I } \otimes \mathbbm {1}_{\mathcal {A}_O \mathcal {B}_O}, \end{aligned}$$

where $\rho _{\mathcal {A}_I \mathcal {B}_I } \in \Omega (\mathcal {A}_I \otimes \mathcal {B}_I)$ is an arbitrary quantum state and $\mathbbm {1}_{\mathcal {A}_O \mathcal {B}_O} \in \text{L}(\mathcal {A}_O \otimes \mathcal {B}_O)$. The set of all process matrices of this form will be denoted by $\mathbf {W^{A || B}}$.

**Figure 1:**
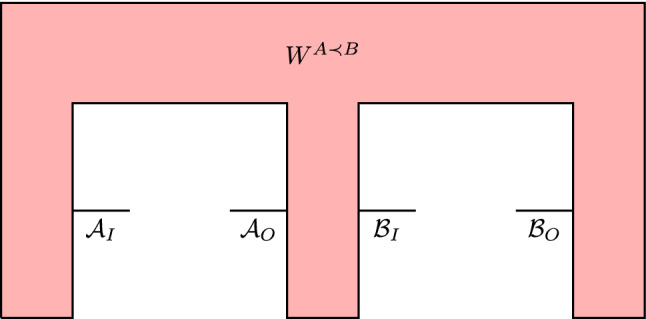
A schematic representation of a process matrix $W^{A \prec B}$ representing a quantum comb $A \prec B$.

We often consider process matrices corresponding to quantum combs^22^. For example, a quantum comb $A \prec B$ (see in Fig. 1) shows that Alice’s and Bob’s operations are performed in causal order. This means that Bob cannot signal to Alice and the choice of Bob’s instrument cannot influence the statistics Alice records. Such process matrices are formally defined in the following way.

### Definition 4

We say that $W^{A \prec B} \in \mathbf {W^{PROC}}$ is a process matrix representing a quantum comb $A \prec B$ if it satisfies the following conditions

17 $$\begin{aligned} \begin{aligned} W^{A \prec B} = W'_{\mathcal {A}_I\mathcal {A}_O\mathcal {B}_I} \otimes \mathbbm {1}_{\mathcal {B}_O}, \\ {{\,\text{tr}\,}}_{\mathcal {B}_I} W'_{\mathcal {A}_I\mathcal {A}_O\mathcal {B}_I} = W''_{\mathcal {A}_I} \otimes \mathbbm {1}_{\mathcal {A}_O}. \end{aligned} \end{aligned}$$

The set of all process matrices of this form will be denoted by $\mathbf {W^{A \prec B}}$.

One can easily observe that the set $\mathbf {W^{A || B}}$ is an intersection of the sets $\mathbf {W^{A \prec B}}$ and $\mathbf {W^{B \prec A}}$. Finally, the definition of the set $\mathbf {W^{A \prec B}}$, together with $\mathbf {W^{B \prec A}}$ allow us to provide their convex hull which is called as causally separable process matrices.

### Definition 5

We say that $W^{SEP} \in \mathbf {W^{PROC}}$ is a causally separable process matrix if it is of the form

18 $$\begin{aligned} W^{\text {SEP}} = p W^{A \prec B } + (1-p) W^{B\prec A}, \end{aligned}$$

where $W^{A \prec B} \in \mathbf {W^{A \prec B}}$, $W^{B\prec A} \in \mathbf {W^{B \prec A}}$ for some parameter $p \in [0,1]$. The set of all causally separable process matrices will be denoted by $\mathbf {W^{SEP}}$.

**Figure 2:**
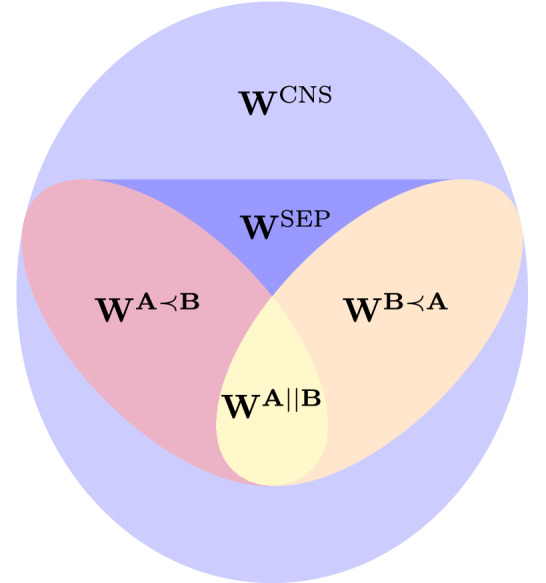
A schematic representation of the sets of process matrices $\mathbf {W^{PROC}}$.

There are however process matrices that do not correspond to a causally separable process and such process matrices are known as causally non-separable (CNS). The examples of such matrices were provided in Ref.^5,31^. The set of all causally non-separable process matrices will be denoted by $\mathbf {W^{CNS}}$. In Fig. 2 we present a schematic plot of the sets of process matrices.

## Discrimination task

This section presents the concept of discrimination between pairs of process matrices. It is worth emphasizing that the definition of a process matrix is a generalization of the concept of quantum states, channels, superchannels^32^ and even generalized supermaps^33,34^. The task of discrimination between process matrices poses a natural extension of discrimination of quantum states^35^, channels^36^ or measurements^13^. The process matrices discrimination task can be described by the following scenario.

Let us consider two process matrices $W_0, W_1 \in \mathbf {W^{PROC}}$. The classical description of process matrices $W_0, W_1$ is assumed to be known to the participating parties. We know that one of the process matrices, $W_0$ or $W_1$, describes the actual correlation between Alice’s and Bob’s laboratories, but we do not know which one. Our aim is to determine, with the highest possible probability, which process matrix describes this correlation. For this purpose, we construct a discrimination strategy *S*. In the general approach, such a strategy *S* is described by an instrument $S = \{ S_0, S_1 \}$. Due to the requirement given by Eq. (14), the instrument *S* must fulfill the condition $S_0 + S_1 \in \textbf{NS}(\mathcal {A}_I \otimes \mathcal {A}_O \otimes \mathcal {B}_I \otimes \mathcal {B}_O)$. The result of composing a process matrix *W* with the discrimination strategy *S* results in a classical label which can take values zero or one. If the label zero occurs, we decide to choose that the correlation is given by $W_0$. Otherwise, we decide to choose $W_1$. In this setting the maximum success probability $p_{\text {succ}} (W_0, W_1)$ of correct discrimination between two process matrices $W_0$ and $W_1$ can be expressed by

19 $$\begin{aligned} p_{\text {succ}} (W_0, W_1) = \frac{1}{2} \max _{S= \{S_0, S_1 \}} \left[ {{\,\text{tr}\,}}(W_0 S_0) + {{\,\text{tr}\,}}(W_1 S_1) \right] . \end{aligned}$$

The following theorem provides the optimal probability of process matrices discrimination as a direct analogue of the Holevo–Helstrom theorem for quantum states and channels.

### Theorem 1

Let $W_0, W_1 \in \mathbf {W^{PROC}}$ be two process matrices. For every choice of discrimination strategy $S = \{ S_0, S_1\}$, it holds that

20 $$\begin{aligned} \begin{aligned}{}&\frac{1}{2} {{\,\text{tr}\,}}(S_0W_0) + \frac{1}{2} {{\,\text{tr}\,}}(S_1W_1) \le \\ {}&\frac{1}{2} + \frac{1}{4 } \max \left\{ \Vert \sqrt{N} (W_0 - W_1) \sqrt{N} \Vert _1: N \in \textbf{NS}(\mathcal {A}_I \otimes \mathcal {A}_O \otimes \mathcal {B}_I \otimes \mathcal {B}_O) \right\} , \end{aligned} \end{aligned}$$

where $\textbf{NS}(\mathcal {A}_I \otimes \mathcal {A}_O \otimes \mathcal {B}_I \otimes \mathcal {B}_O)$ is the set of Choi matrices of non-signaling channels. Moreover, there exists a discrimination strategy *S*, which saturates the inequality Eq. (20).

### Proof

Let us define the sets

21 $$\begin{aligned} \textbf{A} :=\left\{ (S_0, S_1):S_0 + S_1 \in \textbf{NS}(\mathcal {A}_I \otimes \mathcal {A}_O \otimes \mathcal {B}_I \otimes \mathcal {B}_O) ,\,\, S_0, \, S_1 \ge 0 \right\} , \end{aligned}$$

and

22 $$\begin{aligned} \begin{aligned} \textbf{B} :=\{ (\sqrt{N} Q_0 \sqrt{N}, \sqrt{N} Q_1 \sqrt{N}):&\, N \in \textbf{NS}(\mathcal {A}_I \otimes \mathcal {A}_O \otimes \mathcal {B}_I \otimes \mathcal {B}_O), \\ {}&Q_0,\, Q_1 \ge 0, \\ {}&Q_0 + Q_1 = \mathbbm {1}_{\mathcal {A}_I \mathcal {A}_O \mathcal {B}_I\mathcal {B}_O} \}. \end{aligned} \end{aligned}$$

We prove the equality between sets $\textbf{A}$ and $\textbf{B}$. To show $\textbf{B} \subseteq \textbf{A}$, it is suffices to observe that $\sqrt{N} Q_0 \sqrt{N} + \sqrt{N} Q_1 \sqrt{N} \in \textbf{NS}(\mathcal {A}_I \otimes \mathcal {A}_O \otimes \mathcal {B}_I \otimes \mathcal {B}_O)$. To prove $\textbf{A} \subseteq \textbf{B}$ let us take $N :=S_0 + S_1$. It implies that

23 $$\begin{aligned} \Pi _{\text {im} (N)} = \sqrt{N}^{-1} N \sqrt{N}^{-1} = \sqrt{N}^{-1} S_0 \sqrt{N}^{-1} + \sqrt{N}^{-1} S_1 \sqrt{N}^{-1}. \end{aligned}$$

Let us fix $\widetilde{Q_0} :=\sqrt{N}^{-1} S_0 \sqrt{N}^{-1}$ and $\widetilde{Q_1} :=\sqrt{N}^{-1} S_1 \sqrt{N}^{-1}$. Then, we have $\mathbbm {1}_{\mathcal {A}_I \mathcal {A}_O \mathcal {B}_I\mathcal {B}_O} = \mathbbm {1}_{\mathcal {A}_I \mathcal {A}_O \mathcal {B}_I\mathcal {B}_O} - \Pi _{\text {im} (N)} + \Pi _{\text {im} (N)} = \mathbbm {1}_{\mathcal {A}_I \mathcal {A}_O \mathcal {B}_I\mathcal {B}_O} - \Pi _{\text {im} (N)} + \widetilde{Q_0} + \widetilde{Q_1}$. Finally, it is suffices to take

24 $$\begin{aligned} Q_0 :=\mathbbm {1}_{\mathcal {A}_I \mathcal {A}_O \mathcal {B}_I\mathcal {B}_O} - \Pi _{\text {im} (N)} + \widetilde{Q_0}, \end{aligned}$$

and $Q_1 :=\widetilde{Q_1}$. It implies that $\textbf{A} = \textbf{B}$. In conclusion, we obtain

25 $$\begin{aligned} \begin{aligned}{}&\frac{1}{2} {{\,\text{tr}\,}}(S_0W_0) + \frac{1}{2} {{\,\text{tr}\,}}(S_1W_1) \\ {}&= \frac{1}{2} {{\,\text{tr}\,}}\left( \sqrt{N} Q_0 \sqrt{N} W_0 \right) + \frac{1}{2} {{\,\text{tr}\,}}\left( \sqrt{N} Q_1 \sqrt{N} W_1 \right) \\ {}&= \frac{1}{2} {{\,\text{tr}\,}}\left( Q_0 \sqrt{N} W_0 \sqrt{N} \right) + \frac{1}{2} {{\,\text{tr}\,}}\left( Q_1 \sqrt{N} W_1 \sqrt{N} \right) \\ {}&\le \frac{1}{2} + \frac{1}{4 } \max \left\{ || \sqrt{N} (W_0 - W_1) \sqrt{N} ||_1: N \in \textbf{NS}(\mathcal {A}_I \otimes \mathcal {A}_O \otimes \mathcal {B}_I \otimes \mathcal {B}_O) \right\} . \end{aligned} \end{aligned}$$

Moreover, from Holevo–Helstrom theorem^36^ there exists a projective binary measurement $Q= \{ Q_0, Q_1\}$ such that the last inequality is saturated, which completes the proof. $\square$

### Corollary 1

The maximum probability $p_{\text {succ}} (W_0, W_1)$ of correct discrimination between two process matrices $W_0$ and $W_1$ is given by

26 $$\begin{aligned} \begin{aligned}{}&p_{\text {succ}} (W_0, W_1) = \\&\frac{1}{2} + \frac{1}{4 } \max \left\{ \Vert \sqrt{N} (W_0 - W_1) \sqrt{N} \Vert _1: N \in \textbf{NS}(\mathcal {A}_I \otimes \mathcal {A}_O \otimes \mathcal {B}_I \otimes \mathcal {B}_O) \right\} . \end{aligned} \end{aligned}$$

**Figure 3:**
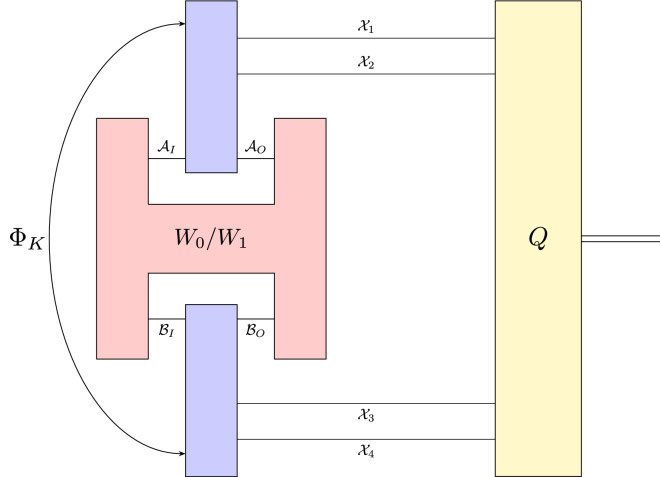
A schematic representation of the setup for distinguishing between process matrices $W_0$ and $W_1$. The discrimination strategy is constructed by using the quantum channel $\Phi _K: \text{L}(\mathcal {A}_I \otimes \mathcal {B}_I) \rightarrow \text{L}(\mathcal {A}_O \otimes \mathcal {B}_O \otimes \mathcal {X}_1 \otimes \mathcal {X}_2 \otimes \mathcal {X}_3 \otimes \mathcal {X}_4 )$ and the binary measurement $Q = \{ Q_0, Q_1\}$ defined in the proof of Theorem 1.

As a valuable by-product of Theorem 1, we receive a realization of process matrices discrimination scheme. The schematic representation of this setup is presented in Fig. 3. To distinguish the process matrices $W_0$ and $W_1$, Alice and Bob prepare the strategy $S = \{ S_0, S_1 \}$ such that $S_0 + S_1 \in \textbf{NS}(\mathcal {A}_I \otimes \mathcal {A}_O \otimes \mathcal {B}_I \otimes \mathcal {B}_O)$. To implement it, let us introduce complex Euclidean spaces $\mathcal {X}_1, \ldots , \mathcal {X}_4$ such that $\dim \left( \bigotimes _{i=1}^4 \mathcal {X}_i \right) = \dim (\mathcal {A}_I \otimes \mathcal {A}_O \otimes \mathcal {B}_I \otimes \mathcal {B}_O)$. Alice and Bob prepare the quantum channel $\Phi _K: \text{L}(\mathcal {A}_I \otimes \mathcal {B}_I) \rightarrow \text{L}(\mathcal {A}_O \otimes \mathcal {B}_O \otimes \mathcal {X}_1 \otimes \mathcal {X}_2 \otimes \mathcal {X}_3 \otimes \mathcal {X}_4 )$ with the Choi matrix *K* given by

27 $$\begin{aligned} K = \left( \mathbbm {1}_{\mathcal {X}_{1,2,3,4}} \otimes \sqrt{N} \right) \left( |\mathbbm {1}\rangle \rangle \langle \langle \mathbbm {1}| \right) \left( \mathbbm {1}_{\mathcal {X}_{1,2,3,4}} \otimes \sqrt{ N }\right) , \end{aligned}$$

where $| \mathbbm {1}\rangle \rangle \in \text{L}(\mathcal {X}_1 \otimes \mathcal {X}_2 \otimes \mathcal {X}_3 \otimes \mathcal {X}_4 \otimes \mathcal {A}_I \otimes \mathcal {A}_O \otimes \mathcal {B}_I \otimes \mathcal {B}_O )$ and $N\in \textbf{NS}(\mathcal {A}_I \otimes \mathcal {A}_O \otimes \mathcal {B}_I \otimes \mathcal {B}_O)$ maximizes the trace norm $\Vert \sqrt{N } (W_0 -W_1) \sqrt{N} \Vert _1$. It is worth noting that the quantum channel $\Phi _K$ is correctly defined due to the fact that ${{\,\text{tr}\,}}_{\mathcal {X}_{1,2,3,4}} K \in \textbf{NS}(\mathcal {A}_I \otimes \mathcal {A}_O \otimes \mathcal {B}_I \otimes \mathcal {B}_O)$. Afterwards, they perform the binary measurement $Q = \{ Q_0, Q_1\}$, where the effect $Q_0 \in \text{L}(\mathcal {X}_1 \otimes \mathcal {X}_2 \otimes \mathcal {X}_3 \otimes \mathcal {X}_4)$ is defined by Eq. (24). Next, they decide which process matrix was used during the calculation assuming $W_0$ if the measurement label is 0. Otherwise, they assume $W_1$.

## Discrimination between different classes of process matrices

This section presents some examples of discrimination between different classes of process matrices. We begin our consideration with the problem of discrimination between two free process matrices $\mathbf {W^{A || B}}$. Next, we will consider various cases of process matrices discrimination representing a quantum comb. First, we calculate exact probability of correct discrimination between two process matrices come from the same class $\mathbf {W^{A \prec B}}$. Next, we study the discrimination task assuming that one of the process matrices is of the form $\mathbf {W^{A \prec B}}$ and the other one is of the form $\mathbf {W^{B \prec A}}$. Finally, we construct a particular class of process matrices which can be perfectly distinguished.

### Free process matrices

The following consideration confirms an intuition that the task of discrimination between free process matrices reduces to the problem of discrimination between quantum states.

From definition of $p_{\text {succ}}(W_0, W_1)$ we have

28 $$\begin{aligned} \begin{aligned} p_{\text {succ}} \left( W_0, W_1\right) = \frac{1}{2} \max _{S=\{S_0, S_1\}} \left[ {{\,\text{tr}\,}}\left( W_0 S_0\right) + {{\,\text{tr}\,}}\left( W_1 S_1\right) \right] . \end{aligned} \end{aligned}$$

Let $W_0$ and $W_1$ be two process matrices of the form $W_0 = \rho \otimes \mathbbm {1}_{\mathcal {A}_O \mathcal {B}_O}$ and $W_1 = \sigma \, \otimes \, \mathbbm {1}_{\mathcal {A}_O \mathcal {B}_O}$, where $\rho , \, \sigma \in \Omega (\mathcal {A}_I \otimes \mathcal {B}_I)$. Then, $p_{\text {succ}}$ is exactly equal to

29 $$\begin{aligned} \begin{aligned}{}&\max _{S= \{ S_0, S_1\}} \left[ \frac{1}{2} {{\,\text{tr}\,}}\left( W_0 S_0\right) +\frac{1}{2} {{\,\text{tr}\,}}\left( W_1 S_1\right) \right] \\&=\quad \max _{S= \{ S_0, S_1\}} \left[ \frac{1}{2} {{\,\text{tr}\,}}\left( (\rho \otimes \mathbbm {1}) S_0 \right) + \frac{1}{2} {{\,\text{tr}\,}}\left( (\sigma \otimes \mathbbm {1}) S_1 \right) \right] \\&= \quad \max _{S= \{ S_0, S_1\}} \left[ \frac{1}{2} {{\,\text{tr}\,}}\left( \rho {{\,\text{tr}\,}}_{\mathcal {A}_O\mathcal {B}_O} S_0 \right) + \frac{1}{2} {{\,\text{tr}\,}}\left( \sigma {{\,\text{tr}\,}}_{\mathcal {A}_O\mathcal {B}_O} S_1 \right) \right] . \end{aligned} \end{aligned}$$

Let us observe ${{\,\text{tr}\,}}_{\mathcal {A}_O\mathcal {B}_O}S_0 + {{\,\text{tr}\,}}_{\mathcal {A}_O\mathcal {B}_O}S_1 = \mathbbm {1}_{\mathcal {A}_I \mathcal {B}_I}$. So, $\{ {{\,\text{tr}\,}}_{\mathcal {A}_O\mathcal {B}_O}S_0, {{\,\text{tr}\,}}_{\mathcal {A}_O\mathcal {B}_O}S_1 \}$ is a binary measurement and therefore, from Holevo–Helstrom theorem for quantum states, we have

30 $$\begin{aligned} \max _{S= \{ S_0, S_1\}} \left[ \frac{1}{2} {{\,\text{tr}\,}}\left( \rho {{\,\text{tr}\,}}_{\mathcal {A}_O\mathcal {B}_O} S_0 \right) + \frac{1}{2} {{\,\text{tr}\,}}\left( \sigma {{\,\text{tr}\,}}_{\mathcal {A}_O\mathcal {B}_O} S_1 \right) \right] \le \frac{1}{2} + \frac{1}{4} \Vert \rho - \sigma \Vert _1. \end{aligned}$$

Now, assume that $E = \{ E_0, E_1 \}$ is the Holevo–Helstrom measurement (by taking $E_0$ and $E_1$ as positive and negative part of $\rho - \sigma$, respectively). Hence, we obtain

31 $$\begin{aligned} \max _{E= \{ E_0, E_1\}} \left[ \frac{1}{2} {{\,\text{tr}\,}}\left( \rho E_0 \right) + \frac{1}{2} {{\,\text{tr}\,}}\left( \sigma E_1 \right) \right] = \frac{1}{2} + \frac{1}{4} \Vert \rho - \sigma \Vert _1. \end{aligned}$$

Observe, it is suffices to take $S_0 :=E_0 \otimes |0\rangle \! \langle 0| \otimes |0\rangle \! \langle 0|$ and $S_1 :=E_1 \otimes |0\rangle \! \langle 0| \otimes |0\rangle \! \langle 0|$. Note that $S_0 + S_1 = \mathbbm {1}_{\mathcal {A}_I \mathcal {B}_I}\otimes |0\rangle \! \langle 0| \otimes |0\rangle \! \langle 0|$ is non-signaling channel. Therefore, we have

32 $$\begin{aligned} p_{\text {succ}} \left( W_0, W_1\right) = \frac{1}{2} + \frac{1}{4} \Vert \rho - \sigma \Vert _1, \end{aligned}$$

which completes the consideration.

Due to the above consideration, we obtain the following corollary.

#### Corollary 2

Let $\rho , \sigma \in \Omega (\mathcal {A}_I \otimes \mathcal {B}_I)$ be quantum states and let $W_0, W_1 \in \mathbf {W^{A || B}}$ be two free process matrices of the form $W_0 = \rho \otimes \mathbbm {1}$ and $W_1=\sigma \otimes \mathbbm {1}$. Then,

33 $$\begin{aligned} p_{\text {succ}} \left( W_0, W_1 \right) = \frac{1}{2} + \frac{1}{4} \Vert \rho - \sigma \Vert _1. \end{aligned}$$

### Process matrices representing quantum combs

Here, we will compare the probability of correct discrimination between two process matrices being quantum combs of the form $W_0^{A \prec B},W_1^{A \prec B }\in \mathbf {W^{A \prec B}}$ by using non-signalling strategy $S = \{ S_0, S_1 \}$ described by Eq. (54) or an adaptive strategy.

**Figure 4:**
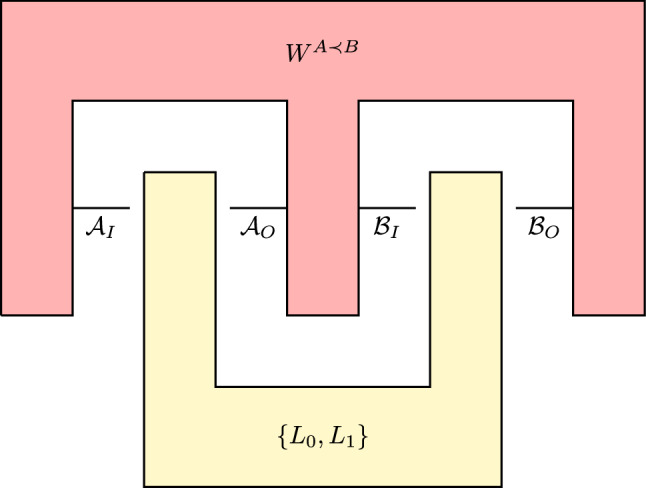
A schematic representation of an adaptive strategy discriminating two process matrices $W_0, W_1 \in W^{A \prec B}$ by using a quantum tester $\{ L_0, L_1\}$.

Before that, we will discuss the issue of adaptive strategy. The most general strategy of quantum operations discrimination is known as an adaptive strategy^22,37^. An adaptive strategy is realized by a quantum tester^1^. A schematic representation of this setup is presented in Fig. 4.

Let us consider a quantum tester $\{ L_0, L_1 \}$ which is a two-element collection of probabilistic quantum networks $L_0$ and $L_1$ whose sum is a quantum comb, and additionally $L_i * W \in \mathbb {R}$, $i =0,1$ for any process matrix $W \in \mathbf {W^{A \prec B}}$. The probability of correct discrimination between $W_0^{A \prec B}$ and $W_1^{A \prec B}$ by using an adaptive strategy is defined by equation

34 $$\begin{aligned} p_{adapt }\left( W_0^{A \prec B}, W_1^{A \prec B} \right) :=\frac{1}{2} \max _{ \{ L_0, L_1\}} L_0 * W_0 + L_1 * W_1. \end{aligned}$$

It turns out that we do not need adaptation in order to obtain the optimal probability os distinction. This is stated formally in the following theorem.

#### Theorem 2

Let $W_0^{A \prec B}, W_1^{A \prec B} \in \mathbf {W^{A \prec B}}$ be two process matrices representing quantum combs $A \prec B$. Then,

35 $$\begin{aligned} p_{succ} \left( W_0^{A \prec B}, W_1^{A \prec B} \right) = p_{adapt }\left( W_0^{A \prec B}, W_1^{A \prec B} \right) . \end{aligned}$$

#### Proof

For simplicity, we will omit superscripts ($A \prec B$ and $B\prec A$). The inequality $p_{succ} \left( W_0, W_1 \right) \le p_{adapt }\left( W_0, W_1 \right)$ is trivial by observing that we calculate maximum value over a larger set.

To show $p_{succ} \left( W_0, W_1 \right) \ge p_{adapt }\left( W_0, W_1 \right)$, let us consider the quantum tester $\{ L_0, L_1\}$ which maximizes Eq. (34), that means

36 $$\begin{aligned} p_{adapt}\left( W_0, W_1\right) = \frac{1}{2} \left( L_0 * W_0 + L_1 * W_1 \right) . \end{aligned}$$

Hence, from definition of $W^{A \prec B}$ we have

37 $$\begin{aligned} W^{A \prec B} = W'\otimes \mathbbm {1}_{\mathcal {B}_O}, \end{aligned}$$

and then we obtain

38 $$\begin{aligned} \frac{1}{2} \left( L_0 * W_0 + L_1 * W_1 \right) = \frac{1}{2} {{\,\text{tr}\,}}\left( W'_0 {{\,\text{tr}\,}}_{\mathcal {B}_O} L_0 + W'_1 {{\,\text{tr}\,}}_{\mathcal {B}_O} L_1 \right) . \end{aligned}$$

Observe that ${{\,\text{tr}\,}}_{\mathcal {B}_O} (L_0 + L_1 ) = \mathbbm {1}_{\mathcal {B}_I} \otimes J$, where *J* is a Choi matrix of a channel $\Phi _J: \text{L}(\mathcal {A}_I) \rightarrow \text{L}(\mathcal {A}_O )$. Let us define a strategy $S = \{S_0, S_1 \}$ such that

39 $$\begin{aligned} \begin{aligned}{}&S_0 = {{\,\text{tr}\,}}_{\mathcal {B}_O} L_0 \otimes \frac{\mathbbm {1}_{\mathcal {B}_O}}{\dim (\mathcal {B}_O)},\\&S_1= {{\,\text{tr}\,}}_{\mathcal {B}_O} L_1 \otimes \frac{\mathbbm {1}_{\mathcal {B}_O}}{\dim (\mathcal {B}_O)},\\&S = S_0 + S_1. \end{aligned} \end{aligned}$$

It easy to observe that $S\in \textbf{NS}(\mathcal {A}_O \otimes \mathcal {A}_I \otimes \mathcal {B}_O \otimes \mathcal {B}_I)$. Then, we have

40 $$\begin{aligned} \frac{1}{2} \left( S_0 * W_0 + S_1 * W_1 \right) = \frac{1}{2} \left( {{\,\text{tr}\,}}\left( W'_0 {{\,\text{tr}\,}}_{\mathcal {B}_O} L_0 + W'_1 {{\,\text{tr}\,}}_{\mathcal {B}_O} L_1 \right) \right) . \end{aligned}$$

It implies that

41 $$\begin{aligned} p_{succ} \left( W_0, W_1\right) \ge p_{adapt }\left( W_0,W_1\right) , \end{aligned}$$

which completes the proof. $\square$

### Process matrices of the form $W^{A \prec B}$ and $W^{B \prec A}$

Now, we present some results for discrimination task assuming the one of the process matrices if of the form $\mathbf {W^{A \prec B}}$ and the other one is of the form $\mathbf {W^{B \prec A}}$. We will construct a particular class of such process matrices for which the perfect discrimination is possible.

**Figure 5:**
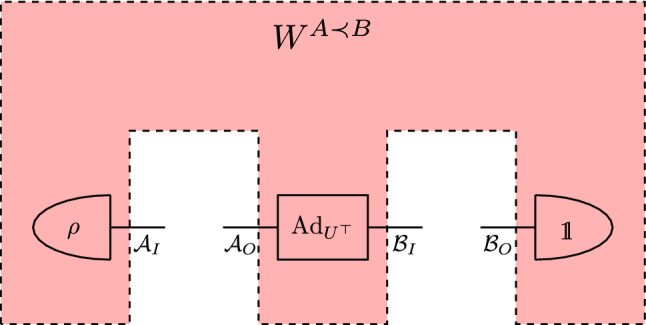
A schematic representation of process matrix $W^{A \prec B}$ given by Eq. (42).

Let us define a process matrix of the form

42 $$\begin{aligned} W^{A \prec B} = \rho \otimes |U \rangle \rangle \langle \langle U | \otimes \mathbbm {1}, \end{aligned}$$

where $\rho \in \Omega (\mathcal {A}_I), |U \rangle \rangle \langle \langle U |$ is the Choi matrix of a unitary channel $\text{Ad}_{U^\top }:\text{L}(\mathcal {A}_O) \rightarrow \text{L}(\mathcal {B}_I)$ of the form $\text{Ad}_{U^\top }(X) = U^\top X \, \bar{U}$ and $\mathbbm {1}\in \text{L}(\mathcal {B}_O)$. A schematic representation of this process matrix we can see in Fig. 5.

#### Proposition 1

Let $W^{A \prec B}$ be a process matrix given by Eq. (42). Let us define a process matrix $W^{ B \prec A }$ of the form

43 $$\begin{aligned} W^{ B \prec A } = P_{\pi } W^{A \prec B} P_{\pi }, \end{aligned}$$

where $P_{\pi }$ is the swap operator replacing the systems $\mathcal {A}_I \rightarrow \mathcal {B}_I$ and $\mathcal {A}_O \rightarrow \mathcal {B}_O$. Then, the process matrix $W^{A \prec B}$ is perfectly distinguishable from $W^{B \prec A}$.

#### Proof

Let us consider the process matrix given by Eq. (42) described by Fig. 5. W.l.o.g. let *d* be a dimension of each of the systems. Let $\rho = \sum _{i=0}^{d-1} \lambda _i |x_i\rangle \! \langle x_i|$, where $\lambda _i \ge 0$ such that $\sum _i \lambda _i = 1$. Based on the spectral decomposition of $\rho$ we create the unitary matrix *V* by taking *i*-th eigenvector of $\rho$, and the measurement $\Delta _V$ (in basis of $\rho$) given by

44 $$\begin{aligned} \Delta _V (X)= \sum _{i=0}^{d-1} \langle x_i| X|x_i \rangle |i\rangle \! \langle i| \otimes |x_i\rangle \! \langle x_i|. \end{aligned}$$

Let us also define the permutation matrix $P_\sigma = \sum _{i=0}^{d-1}|x_{{i +1} \text { mod d} }\rangle \! \langle x_i|$ corresponding to the permutation $\sigma = (0,1,\ldots ,d-1)$.

**Figure 6:**
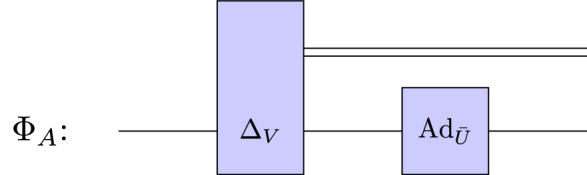
A schematic representation of Alice’s discrimination strategy described by Eq. (45).

Alice and Bob prepare theirs discrimination strategy. Alice performs the local channel (see Fig. 6) given by

45 $$\begin{aligned} \Phi _{A}(\rho ) = \left( \left( \mathcal {I} \otimes \text{Ad}_{\bar{U}} \right) \circ \Delta _V\right) (\rho . \end{aligned}$$

**Figure 7:**
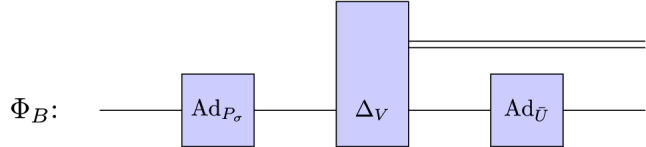
A schematic representation of Bobs’ discrimination strategy described by Eq. (46).

Meanwhile, Bob performs his local channel (see Fig. 7) given by

46 $$\begin{aligned} \Phi _{ B}(\rho ) = \left( \left( \mathcal {I} \otimes \text{Ad}_{\bar{U}} \right) \circ \Delta _V \circ \text{Ad}_{P_\sigma } \right) (\rho ). \end{aligned}$$

$$\square $$

Let us consider the case $A \prec B$. The output after Alice’s action is described by

47 $$\begin{aligned} \Phi _{A}(\rho ) = \sum _{i} \lambda _i |i\rangle \! \langle i| \otimes \bar{U} |x_i\rangle \! \langle x_i| U^\top . \end{aligned}$$

Next, we apply the quantum channel $\text{Ad}_{U^\top }$ (see Fig. 5), and hence we have

48 $$\begin{aligned} (\mathcal {I}\otimes \text{Ad}_{U^\top }) \circ \Phi _{A}(\rho ) = \sum _{i} \lambda _i |i\rangle \! \langle i| \otimes |x_i\rangle \! \langle x_i|. \end{aligned}$$

In the next step, Bob applies his channel as follows

49 $$\begin{aligned} (\mathcal {I} \otimes \Phi _{B}) \circ (\mathcal {I}\otimes \text{Ad}_{U^\top }) \circ \Phi _{A}(\rho ) = \sum _{i} \lambda _i |i\rangle \! \langle i| \otimes |i+1\rangle \! \langle i+1|\otimes |x_{i+1}\rangle \! \langle x_{i+1}|. \end{aligned}$$

Finally, we apply partial trace operation on the subspace $\mathcal {B}_O$ (see Fig. 5), that means

50 $$\begin{aligned} {{\,\text{tr}\,}}_{\mathcal {B}_O} \left( \sum _{i} \lambda _i |i\rangle \! \langle i| \otimes |i+1\rangle \! \langle i+1|\otimes |x_{i+1}\rangle \! \langle x_{i+1}| \right) = \sum _{i} \lambda _i |i\rangle \! \langle i| \otimes |i+1\rangle \! \langle i+1|. \end{aligned}$$

So, the quantum state obtained after the discrimination scenario in the case $A \prec B$ is given by

51 $$\begin{aligned} \sigma ^{A \prec B} = \sum _{i} \lambda _i |i\rangle \! \langle i| \otimes |i+1\rangle \! \langle i+1|. \end{aligned}$$

It implies that if Alice measures her system, she obtains the label *i* with probability $\lambda _i$ whereas Bob obtains the label $(i+ 1) \mod d$ with the same probability. On the other hand, considering the case $B \prec A$, then the state obtained after the discrimination scenario is given by

52 $$\begin{aligned} \sigma ^{B \prec A} = \sum _{i} \lambda _i |i+1\rangle \! \langle i+1| \otimes |i+1\rangle \! \langle i+1|. \end{aligned}$$

So, Bob and Alice obtain the same label $(i+1) \mod d$ with probability $\lambda _i$. Then, the quantum channel $\Phi _K$ (realizing the discrimination strategy *S*) is created as a tensor product of Alice’s and Bob’s local channels, that means $\Phi _K = \Phi _A \otimes \Phi _B$. Due to that they perform the binary measurement $Q= \{ Q_0, Q_1 \}$, where the effect $Q_1$ is given by $Q_1 = \sum _{i=0}^{d-1} |i\rangle \! \langle i| \otimes |i\rangle \! \langle i|$. Hence, we have

53 $$\begin{aligned} p_{\text {succ}}\bigg (W^{A \prec B}, W^{B \prec A}\bigg ) = \frac{1}{2} {{\,\text{tr}\,}}\left( \sigma ^{A \prec B} Q_0\right) + \frac{1}{2} {{\,\text{tr}\,}}\left( \sigma ^{B \prec A} Q_1\right) = 1. \end{aligned}$$

In summary, the process matrices $$W^{A \prec B}$$ and $$W^{B \prec A}$$ are perfectly distinguishable by Alice and Bob which completes the proof. $\square$

## SDP program for calculating the optimal probability of process matrices discrimination

In the standard approach, we would need to compute the probability of correct discrimination between two process matrices $W_0$ and $W_1$. For this purpose, we use the semidefinite programming (SDP). This section presents the SDP program for calculating the optimal probability of discrimination between $W_0$ and $W_1$.

Recall that the maximum value of such a probability can be noticed by

54 $$\begin{aligned} p_{\text {succ}} (W_0, W_1) = \frac{1}{2} \max _{S= \{S_0, S_1 \}} \left[ {{\,\text{tr}\,}}(W_0 S_0) + {{\,\text{tr}\,}}(W_1 S_1) \right] , \end{aligned}$$

with requirement that the optimal strategy $S = \{S_0, S_1 \}$ is a quantum instrument such that $S_0 + S_1 \in \textbf{NS}(\mathcal {A}_I \otimes \mathcal {A}_O \otimes \mathcal {B}_I \otimes \mathcal {B}_O)$. Hence, we arrive at the primal and dual problems presented in the Program 1.

To optimize this problem we used the Julia programming language along with quantum package QuantumInformation.jl^38^ and SDP optimization via SCS solver^39,40^ with absolute convergence tolerance $10^{-5}$. The code is available on GitHub^41^.

It may happen that the values of primal and dual programs are equal. This situation is called strong duality. Slater’s theorem provides the set of conditions which guarantee strong duality^36^. It can be shown that Program 1 fulfills conditions of Slater’s theorem (it is suffices to take $Y_0,Y_1 = 0$ and $\alpha > \frac{1}{2} \max \{ \Lambda ^{\text {max}}(W_0), \Lambda ^{\text {max}}(W_1) \}$, where $\Lambda ^{\text {max}}(X)$ is the maximum eigenvalue of *X*). Therefore, we can consider the primal and the dual problem equivalently.

**Table:** Program 1 Semidefinite program for maximizing the probability of correct discrimination between two process matrices $W_0$ and $W_1$

SDP program for calculating the optimal probability of discrimination between $W_0$ and $W_1$	
Primal problem	
maximize:	$\frac{1}{2} {{\,\text{Tr}\,}}(W_0S_0) + \frac{1}{2}{{\,\text{Tr}\,}}(W_1S_1)$
subject to:	${{\,\text{tr}\,}}_{\mathcal {A}_O} (S_0 + S_1) = \frac{\mathbbm {1}_{\mathcal {A}_I}}{\dim (\mathcal {A}_I)} \otimes {{\,\text{tr}\,}}_{\mathcal {A}_O\mathcal {A}_I} (S_0 + S_1),$
	$${{\,\text{tr}\,}}_{\mathcal {B}_O} (S_0 + S_1) = \frac{\mathbbm {1}_{\mathcal {B}_I}}{\dim (\mathcal {B}_I)} \otimes {{\,\text{tr}\,}}_{\mathcal {B}_O\mathcal {B}_I} (S_0 + S_1),$$
	$${{\,\text{tr}\,}}(S_0 + S_1) = \dim (\mathcal {A}_I)\dim (\mathcal {B}_I),$$
	$$S_0 \in \text{Pos}(\mathcal {A}_I \otimes \mathcal {A}_O \otimes \mathcal {B}_I \otimes \mathcal {B}_O),$$
	$$S_1 \in \text{Pos}(\mathcal {A}_I \otimes \mathcal {A}_O \otimes \mathcal {B}_I \otimes \mathcal {B}_O).$$

Dual problem	
minimize:	$\alpha \cdot \dim (\mathcal {A}_I)\dim (\mathcal {B}_I)$
subject to:	$\mathbbm {1}_{\mathcal {A}_O} \otimes Y_0 - \frac{\mathbbm {1}_{\mathcal {A}_I\mathcal {A}_O}}{\dim (\mathcal {A}_I)} \otimes {{\,\text{tr}\,}}_{\mathcal {A}_I}(Y_0) +\mathbbm {1}_{\mathcal {B}_O} \otimes Y_1 +$
	$$- \frac{\mathbbm {1}_{\mathcal {B}_I\mathcal {B}_O}}{\dim (\mathcal {B}_{I})} \otimes {{\,\text{tr}\,}}_{\mathcal {B}_I}Y_1 + \alpha \cdot \mathbbm {1}_{\mathcal {A}_I\mathcal {A}_O\mathcal {B}_I\mathcal {B}_O} \ge \frac{1}{2} W_0,$$
	$$\mathbbm {1}_{\mathcal {A}_O} \otimes Y_0 - \frac{\mathbbm {1}_{\mathcal {A}_I\mathcal {A}_O}}{\dim (\mathcal {A}_I)} \otimes {{\,\text{tr}\,}}_{\mathcal {A}_I}(Y_0) + \mathbbm {1}_{\mathcal {B}_O} \otimes Y_1 +$$
	$$- \frac{\mathbbm {1}_{\mathcal {B}_I\mathcal {B}_O}}{\dim (\mathcal {B}_{I})} \otimes {{\,\text{tr}\,}}_{\mathcal {B}_I}Y_1 + \alpha \cdot \mathbbm {1}_{\mathcal {A}_I\mathcal {A}_O\mathcal {B}_I\mathcal {B}_O} \ge \frac{1}{2} W_1,$$
	$$Y_0 \in \text { Herm}( \mathcal {A}_I \otimes \mathcal {B}_I \otimes \mathcal {B}_O),$$
	$$Y_1 \in \text { Herm}(\mathcal {A}_I \otimes \mathcal {A}_O \otimes \mathcal {B}_I),$$
	$$\alpha \in \mathbb {R}.$$

We can observe a connection between Program 1 and the semidefinite program calculating the maximal probability of successful discrimination of two qubit-qubit quantum channels using two copies under different strategies ^7^. Thanks to their idea, we obtain the following SDP program.

**Table:** 

Primal problem	
given:	$W_0, W_1 \in \mathbf {W^{PROC}}$
maximize:	$\frac{1}{2} {{\,\text{Tr}\,}}(W_0S_0) + \frac{1}{2}{{\,\text{Tr}\,}}(W_1S_1)$
subject to:	$\{ S_0, S_1\}$ is a tester such that
	$$S_0 + S_1 \in \textbf{NS}(\mathcal {A}_I \otimes \mathcal {A}_O \otimes \mathcal {B}_I \otimes \mathcal {B}_O).$$

Dual problem	
given:	$W_0, W_1 \in \mathbf {W^{PROC}}$
minimize:	$\alpha$
subject to:	$\frac{1}{2} W_i \le \alpha \cdot X, \, \text { where } X \in \widetilde{ \textbf{NS}}(\mathcal {A}_I \otimes \mathcal {A}_O \otimes \mathcal {B}_I \otimes \mathcal {B}_O),$
	and $\widetilde{ \textbf{NS}}(\mathcal {A}_I \otimes \mathcal {A}_O \otimes \mathcal {B}_I \otimes \mathcal {B}_O)$ lies in the dual affine
	space of the set $\textbf{NS}(\mathcal {A}_I \otimes \mathcal {A}_O \otimes \mathcal {B}_I \otimes \mathcal {B}_O)$ and $i \in \{0,1\}$.

It can be shown, eg. in^7^ that $\widetilde{ \textbf{NS}}(\mathcal {A}_I \otimes \mathcal {A}_O \otimes \mathcal {B}_I \otimes \mathcal {B}_O)$ creates the set of all process matrices $\mathbf {W^{PROC}}$. Hence, the constrains $\frac{1}{2} W_i \le \alpha X,$ where $X \in \mathbf {W^{PROC}}$, can be explicitly written as in Program 1.

The SDP Program 1 is defined on the tensor product of four Hilbert spaces with local dimensions equal *d*. The number of variables of the primal and dual problem scales as $\Omega \left( d^8 \right)$ and $\Omega \left( d^6 \right)$, respectively, therefore, the standard SDP solvers can take a long time to find the solution for large *d*. One approach to deal with this problem is to use a simplified SDP programming approach like it was done for computing the diamond norm^42^. Another method is to exploit an iterative method based on the fixed point and the Complementary Slackness criterion, like in Ref.^43^ for the fidelity function. We leave this problem for future research.

## Distance between process matrices

In this section we present the semidefinite programs for calculating the distance in trace norm between a given process matrix $W \in \mathbf {W^{PROC}}$ and different subsets of process matrices, such that $\mathbf {W^{A || B}}$ , $\mathbf {W^{A \prec B}}$, $\mathbf {W^{B \prec A}}$ or $\mathbf {W^{SEP}}$.

For example, let us consider the case $\mathbf {W^{A || B}}$. Theoretically, the distance between a process matrix *W* and the set of free process matrices $\mathbf {W^{A || B}}$ can be expressed by

55 $$\begin{aligned} \begin{aligned}{}&\text {dist}\left( W, \mathbf {W^{A || B}} \right) = \\&\min _{\widetilde{W} \in \mathbf {W^{A || B}}} \max \left\{ \Vert \sqrt{N} (W - \widetilde{W}) \sqrt{N} \Vert _1: N \in \textbf{NS}(\mathcal {A}_I \otimes \mathcal {A}_O \otimes \mathcal {B}_I \otimes \mathcal {B}_O) \right\} . \end{aligned} \end{aligned}$$

Analogously, for the sets $\mathbf {W^{A \prec B}}, \mathbf {W^{B \prec A}}$ and $\mathbf {W^{SEP}}$ with the minimization condition $\min _{\widetilde{W} \in \mathbf {W^{A \prec B}}}$, $\min _{\widetilde{W} \in \mathbf {W^{B \prec A}}}$, $\min _{\widetilde{W} \in \mathbf {W^{SEP}}}$, respectively. Due to the results obtained from the previous section (see Program 1) and Slater theorem we are able to note the Eq. (55) to SDP problem presented in the Program 2. We use the SDP optimization via SCS solver^39,40^ with absolute convergence tolerance $10^{-8}$ and relative convergence tolerance $10^{-8}$. The implementations of SDPs in the Julia language are available on GitHub^41^.

**Table:** Semidefinite program for computation the distance between a process matrix *W* and $\Upsilon$, which can be one of the set $\mathbf {W^{A || B }}, \mathbf {W^{A \prec B}}, \mathbf {W^{B \prec A }}$ or $\mathbf {W^{SEP}}$. Depending on the selected set we include additional constrains to SDP described by Eq. (16) for $\mathbf {W^{A || B }}$, Eq. (17) for $\mathbf {W^{A \prec B}}$ and $\mathbf {W^{B \prec A}}$ or Eq. (18) for $\mathbf {W^{SEP}}$

SDP calculating the distance between a process matrix *W* and the set $\Upsilon$.	
minimize:	4 $\dim (\mathcal {A}_I)\dim (\mathcal {B}_I) \alpha -2$
subject to:	$\mathbbm {1}_{\mathcal {A}_O} \otimes Y_0 - \frac{\mathbbm {1}_{\mathcal {A}_I\mathcal {A}_O}}{\dim (\mathcal {A}_I)} \otimes {{\,\text{tr}\,}}_{\mathcal {A}_I}(Y_0) + \mathbbm {1}_{\mathcal {B}_O} \otimes Y_1 +$
	- $\frac{\mathbbm {1}_{\mathcal {B}_I\mathcal {B}_O}}{\dim (\mathcal {B}_{I})} \otimes {{\,\text{tr}\,}}_{\mathcal {B}_I}Y_1 + \alpha \cdot \mathbbm {1}_{\mathcal {A}_I\mathcal {A}_O\mathcal {B}_I\mathcal {B}_O} \ge \frac{1}{2} W,$
	$$\mathbbm {1}_{\mathcal {A}_O} \otimes Y_0 - \frac{\mathbbm {1}_{\mathcal {A}_I\mathcal {A}_O}}{\dim (\mathcal {A}_I)} \otimes {{\,\text{tr}\,}}_{\mathcal {A}_I}(Y_0) +\mathbbm {1}_{\mathcal {B}_O} \otimes Y_1 +$$
	- $\frac{\mathbbm {1}_{\mathcal {B}_I\mathcal {B}_O}}{\dim (\mathcal {B}_{I})} \otimes {{\,\text{tr}\,}}_{\mathcal {B}_I}Y_1 + \alpha \cdot \mathbbm {1}_{\mathcal {A}_I\mathcal {A}_O\mathcal {B}_I\mathcal {B}_O} \ge \frac{1}{2}W^{*},$
	$$\hbox {W}^{*} \in \Upsilon ,$$
	$$Y_0 \in \text { Herm}( \mathcal {A}_I \otimes \mathcal {B}_I \otimes \mathcal {B}_O),$$
	$$Y_1 \in \text { Herm}(\mathcal {A}_I \otimes \mathcal {A}_O \otimes \mathcal {B}_I),$$
	$$\alpha \in \mathbb {R}.$$

### Example

Let $\mathcal {A}_I=\mathcal {A}_O=\mathcal {B}_I=\mathcal {B}_O=\mathbb {C}^2$. Let us consider a causally non-separable process matrix comes from Ref.^5^ of the form

56 $$\begin{aligned} W^{\text {CNS}} = \frac{1}{4} \left[ \mathbbm {1}_{\mathcal {A}_I\mathcal {A}_O\mathcal {B}_I\mathcal {B}_O} + \frac{1}{\sqrt{2}} \left( \sigma _z^{\mathcal {A}_O} \sigma _z^{\mathcal {B}_I} \otimes \mathbbm {1}_{\mathcal {A}_I\mathcal {B}_O} + \sigma _z^{\mathcal {A}_I} \sigma _x^{\mathcal {B}_I} \sigma _z^{\mathcal {B}_O} \otimes \mathbbm {1}_{\mathcal {A}_O} \right) \right] , \end{aligned}$$

where $\sigma _x^\mathcal {X}, \sigma _z^\mathcal {X}$ are Pauli matrices on space $\text{L}(\mathcal {X})$. We have calculated the distance in trace norm between $W^{\text {CNS}}$ and different subset of process matrices. Finally, we obtain

57 $$\begin{aligned}{} & {} \text {dist}\left( W^{\text {CNS}},\mathbf {W^{A || B }}\right) \approx 1.00000001\approx 1, \end{aligned}$$

58 $$\begin{aligned}{} & {} \text {dist}\left( W^{\text {CNS}}, \mathbf {W^{A \prec B }} \right) \approx 0.7071068 \approx \frac{\sqrt{2}}{2}, \end{aligned}$$

59 $$\begin{aligned}{} & {} \text {dist}\left( W^{\text {CNS}}, \mathbf {W^{B \prec A }} \right) \approx 0.7071068 \approx \frac{\sqrt{2}}{2}, \end{aligned}$$

60 $$\begin{aligned}{} & {} \text {dist}\left( W^{\text {CNS}}, \mathbf {W^{SEP }} \right) \approx 0.2928932 \approx 1-\frac{\sqrt{2}}{2}. \end{aligned}$$

**Figure 8:**
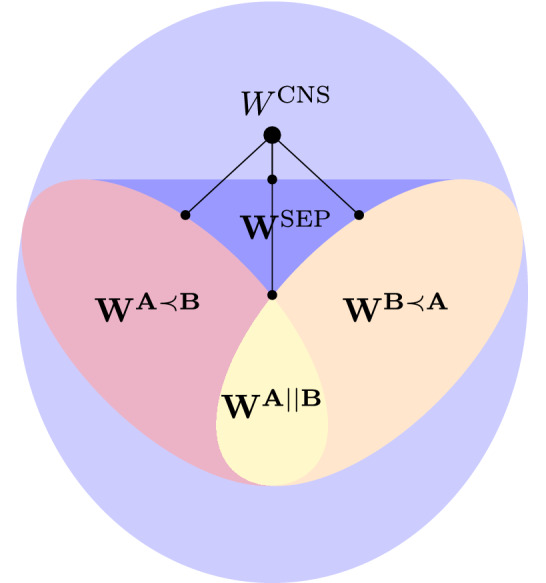
A schematic representation of the distances between $W^{\text {CNS}}$ defined in Eq. (56) and the sets $\mathbf {W^{A || B }}$, $\mathbf {W^{A \prec B }}$, $\mathbf {W^{B \prec A }}$ and $\mathbf {W^{SEP}}$.

The numerical computations give us some intuition about the geometry of the set of process matrices. Those results are presented in Fig. 8. Moreover, by using $W^{\text {CNS}}$ given by Eq. (56) it can be shown that the set of all causally non-separable process matrices is not convex. To show this fact, it suffices to observe that for every $\sigma _i, \sigma _j, \sigma _k, \sigma _l \in \{ \sigma _x, \sigma _y, \sigma _z, \mathbbm {1}\}$ the following equation holds

61 $$\begin{aligned} \left( \sigma _i \otimes \sigma _j \otimes \sigma _k \otimes \sigma _l \right) W^{\text {CNS}} ( \sigma _i \otimes \sigma _j \otimes \sigma _k \otimes \sigma _l)^\dagger \in \mathbf {W^{CNS}}. \end{aligned}$$

Simultaneously, the average of the process matrices of the form Eq. (61) distributed uniformly states $\frac{1}{4} \mathbbm {1}_{\mathbb {C}^{16}}$, however $\frac{1}{4}\mathbbm {1}_{{\mathbb {C}}^{16}} \not \in {\textbf{W}}^{CNS}$. It implies that the set $\textbf{W}^{CNS}$ is not convex.

## Convex cone structure theory

From geometrical point of view, we present an alternative way to derive of Eq. (26). It turns out that the task of process matrices discrimination is strictly connected with the convex cone structure theory. To keep this work self-consistent, the details of convex cone structure theory are presented in the Supplementary Materials.

Let $\mathcal {V}$ be a finite dimensional real vector space with a proper cone $\mathcal {C}\subset \mathcal {V}$. A base *B* of the proper cone $\mathcal {C}$ is a compact convex subset $B \subset \mathcal {C}$ such that each nonzero element $c \in \mathcal {C}$ has a unique representation in the form $c = \alpha \cdot b$, where $\alpha > 0$ and $b \in B$. The corresponding base norm in $\mathcal {V}$ is defined by

62 $$\begin{aligned} ||x||_{B} = \{ \alpha + \beta , x = \alpha b_{1} - \beta b_{2}, \alpha , \beta \ge 0, b_{1}, b_{2} \in B \}. \end{aligned}$$

From Ref.^37^, Corollary 2 the author showed that the base norm can be written as

63 $$\begin{aligned} || x ||_{B} = \sup _{{\widetilde{b}}\in {\widetilde{B}}} ||{\widetilde{b}}^{1/2} x \widetilde{b}^{1/2} ||_{1}, \end{aligned}$$

where

64 $$\begin{aligned} {\widetilde{B}} :=\{ Y \in \mathcal {C}: {{\,\text{tr}\,}}\left( XY \right) = 1, \forall X \in B \}. \end{aligned}$$

### Convex cone structure of process matrices set

Let $\mathcal {V}$ be a Hilbert space given by

65 $$\begin{aligned} \mathcal {V}= \text{Herm}(\mathcal {A}_I\otimes \mathcal {A}_O \otimes \mathcal {B}_I \otimes \mathcal {B}_O), \end{aligned}$$

with proper cone

66 $$\begin{aligned} \mathcal {C}= \{ W \in \mathcal {V}: W \in \text{Pos}(\mathcal {A}_I\otimes \mathcal {A}_O \otimes \mathcal {B}_I \otimes \mathcal {B}_O) \}. \end{aligned}$$

Consider the linear subspace $\mathcal {S}\subset \mathcal {V}$ given by

67 $$\begin{aligned} \begin{aligned} \mathcal {S}= \{W \in \mathcal {V}:&^{}_{B_IB_O}{W} = ^{}_{A_OB_IB_O}{W}, \\&^{}_{A_IA_O}{W} = ^{}_{A_OA_IB_O}{W}, \\&^{}_{A_OB_O}{W} = ^{}_{B_O}{W} + ^{}_{A_O}{W} - W \}, \end{aligned} \end{aligned}$$

together with its proper cone $\mathcal {C}_\mathcal {S}$. Observe that if we fix trace of $W \in \mathcal {C}_\mathcal {S}$ such that ${{\,\text{tr}\,}}(W) = \dim (\mathcal {A}_O) \cdot \dim (\mathcal {B}_O)$, we achieve the set of all process matrices $\mathbf {W^{PROC}}$. And then, $\mathbf {W^{PROC}}$ is a base of $\mathcal {C}_\mathcal {S}$.

#### Proposition 2

Let $\mathbf {W^{PROC}}$ be the set of process matrices. Then, the set $\widetilde{\mathbf {W^{PROC}}}$ is determined by

68 $$\begin{aligned} \widetilde{\mathbf {W^{PROC}}} = \textbf{NS}(\mathcal {A}_I\otimes \mathcal {A}_O \otimes \mathcal {B}_I \otimes \mathcal {B}_O). \end{aligned}$$

#### Proof

To prove this proposition, we need to show that

69 $$\begin{aligned} {{\,\text{tr}\,}}\left( X W \right) = 1 \,\, \text {for all } W \in \mathbf {W^{PROC}} \iff X \in \textbf{NS}(\mathcal {A}_I\otimes \mathcal {A}_O \otimes \mathcal {B}_I \otimes \mathcal {B}_O). \end{aligned}$$

This equivalence, along with its proof, can be found eg. in Refs.^29,44^. However, to keep this work self-consistent we present our modified version of their reasoning.

Let us first take $X \in \textbf{NS}(\mathcal {A}_I\otimes \mathcal {A}_O \otimes \mathcal {B}_I \otimes \mathcal {B}_O)$. Then, from Ref.^25^, Lemma 1, we note

70 $$\begin{aligned} X = \sum _{i} \lambda _i A_i \otimes B_i, \end{aligned}$$

where $A_i \in \textbf{NS}(\mathcal {A}_I\otimes \mathcal {A}_O)$, $B_i \in \textbf{NS}(\mathcal {B}_I\otimes \mathcal {B}_O)$ and $\lambda _i \in \mathbb {R}$ such that $\sum _{i} \lambda _i = 1$. From definition of process matrix and linearity we obtain

71 $$\begin{aligned} {{\,\text{tr}\,}}\left( X W \right) = {{\,\text{tr}\,}}\left( \sum _i \lambda _i (A_i \otimes B_i) W \right) = \sum _i \lambda _i {{\,\text{tr}\,}}\left( ( A_i \otimes B_i ) W \right) = \sum _{i} \lambda _i = 1. \end{aligned}$$

To prove opposite implication, let us take $W = \mathbbm {1}_{\mathcal {A}_O} \otimes J$, where *J* is the Choi matrix of quantum channel $\Phi _J: \text{L}(\mathcal {B}_I \otimes \mathcal {B}_O) \rightarrow \text{L} (\mathcal {A}_I)$. Then, we have

72 $$\begin{aligned} 1={{\,\text{tr}\,}}\left( W X \right) = {{\,\text{tr}\,}}\left( (\mathbbm {1}_{\mathcal {A}_O} \otimes J) X \right) = {{\,\text{tr}\,}}\left( J {{\,\text{tr}\,}}_{\mathcal {A}_O} X \right) . \end{aligned}$$

From Ref.^45^, we have

73 $$\begin{aligned} {{\,\text{tr}\,}}_{\mathcal {A}_O} X = \mathbbm {1}_{\mathcal {A}_I} \otimes P, \end{aligned}$$

where $P \in \text{Pos}(\mathcal {B}_I \otimes \mathcal {B}_O)$. Similarly, if we take $W :=\mathbbm {1}_{\mathcal {B}_O} \otimes K$, where *K* is the Choi matrix of a quantum channel $\Phi _K: \text{L}(\mathcal {A}_I \otimes \mathcal {A}_O) \rightarrow \text{L} (\mathcal {B}_I)$, we obtain

74 $$\begin{aligned} {{\,\text{tr}\,}}_{\mathcal {B}_O} X = \mathbbm {1}_{\mathcal {B}_I} \otimes P, \end{aligned}$$

where $P \in \text{Pos}(\mathcal {A}_I \otimes \mathcal {A}_O)$. It implies that $X \in \textbf{NS}(\mathcal {A}_I\otimes \mathcal {A}_O \otimes \mathcal {B}_I \otimes \mathcal {B}_O)$, which completes the proof. $\square$

Due to Proposition 2, and the fact that the base norm can be also written using the trace norm, like in Eq. (63), we immediately obtain the following corollary.

#### Corollary 3

The base norm $||\cdot ||_\mathbf {W^{PROC}}$ between two process matrices $W_1, W_2 \in \mathbf {W^{PROC}}$ can be expressed as

75 $$\begin{aligned} || W_1 - W_2 ||_{\mathbf {W^{PROC}} } = \max \{ ||\sqrt{N} (W_1 - W_2) \sqrt{N} ||_1: N \in \textbf{NS}(\mathcal {A}_I\otimes \mathcal {A}_O \otimes \mathcal {B}_I \otimes \mathcal {B}_O) \}. \end{aligned}$$

## Conclusion and discussion

In this work, we studied the problem of single shot discrimination between process matrices. Our aim was to provide an exact expression for the optimal probability of correct distinction and quantify it in terms of the trace norm. This value was maximized over all Choi operators of non-signaling channels and and poses direct analogues to the Holevo–Helstrom theorem for quantum channels. In addition, we have presented an alternative way to achieve this expression by using the convex cone structure theory. As a valuable by-product, we have also found the optimal realization of the discrimination task for process matrices that use such non-signalling channels. Additionally, we expressed the discrimination task as semidefinite programming (SDP). Due to that, we have created SDP calculating the distance between process matrices and we expressed it in terms of the trace norm. Moreover, we found an analytical result for discrimination of free process matrices. It turns out that the task of discrimination between free process matrices can be reduced to the task of discrimination between quantum states. Next, we consider the problem of discrimination for process matrices corresponding to quantum combs. We have studied which strategy, adaptive or non-signalling, should be used during the discrimination task. We proved that no matter which strategy you choose, the optimal probability of distinguishing two process matrices being a quantum comb is the same. So, it turned out that we do not need to use some unknown additional processing in this case. Finally, we discovered a particular class of process matrices having opposite causal order, which can be distinguished perfectly. This work paves the way toward a complete description of necessary and sufficient criterion for perfect discrimination between process matrices. Moreover, it poses a starting point to fully describe the geometry of the set of process matrices, particularly causally non-separable process matrices.

## Supplementary Information

Supplementary Information.

## Data Availability

The code used to generate numerical results analyzed during the current study is available in the Github repository, https://github.com/iitis/strategies_for_single_shot_discrimination_of_process_matrices.
