# Supplementary material for: Strategies for single-shot discrimination of process matrices
Source: Sci Rep. 2023 Feb 21;13:3046. doi: 10.1038/s41598-023-30191-0 (PMC9944257; doi:10.1038/s41598-023-30191-0)
Supplement: Supplementary file 1 — Supplementary Information. [file 41598_2023_30191_MOESM1_ESM.pdf]

STRATEGIES FOR SINGLE-SHOT DISCRIMINATION OF PROCESS MATRICES – SUPPLEMENTARY MATERIALS

PAULINA LEWANDOWSKA* [1], ŁUKASZ PAWELA[1], AND ZBIGNIEW PUCHAŁA[1]

[1] *Institute of Theoretical and Applied Informatics, Polish Academy of Sciences, ul. Bałtycka 5, 44-100 Gliwice, Poland*

* E-mail address: plewandowska@iitis.pl

Appendix A. Convex cone structures

To keep this work self-consistent we present in this appendix basic definitions and properties about convex cone structure theory.

Suppose $\mathcal{X}$ is a finite dimensional real vector space and $\mathcal{C} \subset \mathcal{X}$ is a closed convex cone. We assume that $\mathcal{C}$ is pointed, that means $\mathcal{C} \cap -\mathcal{C} = \{0\}$. A closed pointed convex cone is in one-to-one correspondence with partial order in $\mathcal{X}$, by $x \geq y \iff x - y \in \mathcal{C}$ for each $x, y \in \mathcal{X}$. If we additionally assume that the cone is generating, that is for each $x \in \mathcal{X}$ there exists $u, w \in \mathcal{C}$ such that $x = u - w$, then a nonempty set $\mathcal{C} \subseteq \mathcal{X}$ satisfying all above properties will be called a proper cone in space $\mathcal{X}$. Let $\mathcal{X}^*$ be a dual space with duality $\langle \cdot | \cdot \rangle$. Then, we introduce a partial order in $\mathcal{X}^*$ as well with dual cone $\mathcal{C}^* = \{f \in \mathcal{X}^* : \langle f|z \rangle \geq 0, \forall z \in C\}$. Observe that the cone $\mathcal{C}^*$ is also closed and convex. Moreover, if $\mathcal{C}$ is generating in space $\mathcal{X}$, then $\mathcal{C}^*$ is pointed, so we can introduce the partial order in $\mathcal{X}^*$ given by

$$f \geq g \iff f - g \in \mathcal{C}^* \tag{1}$$

for all $f, g \in \mathcal{X}^*$.

Next, consider a linear space with fixed inner product. If $\mathcal{X}$ is an inner product space, then the Riesz representation theorem [1] holds that the inner product determines an isomorphism between $\mathcal{X}$ and $\mathcal{X}^*$. Therefore, the cone $\mathcal{C}$ is equal to $\mathcal{C}^*$.

An interior point $e \in \text{int}(\mathcal{C})$ of a cone $\mathcal{C}$ is called an order unit if for each $x \in \mathcal{X}$, there exists $\lambda > 0$ such that $\lambda e - x \in \mathcal{C}$. Whereas, a base of $\mathcal{C}$ is defined as compact and convex subset $B \subset \mathcal{C}$ such that for every $z \in \mathcal{C} \setminus \{0\}$, there exists unique $t > 0$ and an element $b \in B$ such that $z = tb$. It can be shown that the set

$$B = \{z \in \mathcal{C} : \langle e|z \rangle = 1\} \tag{2}$$

is the base of $\mathcal{C}$ (determined by element e) if and only if an element e is an order unit and $e \in \text{int}\,(\mathcal{C}^*)$. Finally, we define the base norm as

$$||x||_B = \{\alpha + \beta, x = \alpha b_1 - \beta b_2, \alpha, \beta \geq 0, b_1, b_2 \in B\}. \tag{3}$$

It can be shown [2] that the base norm is expressed as

$$||x||_B = \sup_{\widetilde{b}\in\widetilde{B}} ||\widetilde{b}^{1/2} x \widetilde{b}^{1/2}||_1, \tag{4}$$

where $\widetilde{B} = \{\widetilde{b} \in \mathcal{C} : \mathrm{tr}(b\widetilde{b}) = 1, \forall b \in B\}$.

References

[1] W. Rudin, "Functional analysis 2nd ed," *International Series in Pure and Applied Mathematics. McGraw-Hill, Inc., New York*, 1991.

[2] A. Jenčová, "Base norms and discrimination of generalized quantum channels," *Journal of Mathematical Physics*, vol. 55, no. 2, p. 022201, 2014.
